# A Polydopamine-Coated Platinum Nanoplatform for Tumor-Targeted Photothermal Ablation and Migration Inhibition

**DOI:** 10.3389/fonc.2022.860718

**Published:** 2022-03-03

**Authors:** Xianwen Zou, Guiqi Ma, Pengyu Zhu, Yutao Cao, Xiao Sun, Haijun Wang, Jian Dong

**Affiliations:** ^1^Institute of Optical Functional Materials for Biomedical Imaging, School of Chemistry and Pharmaceutical Engineering, Shandong First Medical University and Shandong Academy of Medical Sciences, Taian, China; ^2^School of Life Sciences, Shandong First Medical University and Shandong Academy of Medical Sciences, Taian, China

**Keywords:** platinum nanoplatform, RGD target, PTT, photothermal ablation, migration inhibition

## Abstract

In this work, Arg-Gly-Asp (RGD) peptide-coupled polydopamine-modified mesoporous platinum nanoparticles (mPt@PDA-RGD NPs) were developed for targeted photothermal therapy (PTT) and migration inhibition of SKOV-3 cells. mPt@PDA-RGD NPs with obvious core/shell structure demonstrated high photothermal performance under 808-nm near-infrared (NIR) laser irradiation. mPt@PDA-RGD NPs with favorable biocompatibility exhibited remarkable SKOV-3 inhibition ability under NIR laser irradiation. Moreover, compared to mPt@PDA NPs, the RGD-functionalized NPs achieved more tumor uptake and PTT performance, which was attributed to the specific interaction between RGD of NPs and α_v_β_3_ integrin overexpressed by SKOV-3. Importantly, cell scratch experiments indicated that the photothermal effect of mPt@PDA-RGD NPs can effectively inhibit the migration of surviving SKOV-3 cells, which was assigned to disturbance of the actin cytoskeleton of SKOV-3. Thus, mPt@PDA-RGD NPs presented great potential for targeted tumor photothermal ablation and migration inhibition.

## Introduction

Tumor has emerged as a serious threat to human health. As a popular noninvasive treatment for local tumors, photothermal therapy (PTT) has attracted great attention worldwide (1–4). PTT usually depends on the photosensitizer to create enough heat to inhibit tumor or even kill them and has been recognized as an effective and minimally invasive treatment strategy for primary tumors (5, 6). Compared to traditional treatments of cancer, such as radiotherapy and chemotherapy, PTT has certain advantages including less invasiveness, fewer side effects, and higher specificity (7, 8). The photosensitizer is expected to be nontoxic and relatively safe to cells in the absence of any laser irradiation. In addition, due to the precise control of laser irradiation parameters (such as position, laser wavelength, irradiation time, and light intensity), PTT can show great selectivity and minimal side effects (9, 10).

The development of nanomedicine improves the diagnosis and treatment efficiency of tumors (11–14). Nanomaterials with plasmonic nanostructure can be well activated by near-infrared (NIR) for PTT of cancer (15, 16). For example, gold nanoparticles (GNPs) have attracted much attention due to their high photothermal conversion efficiency (17, 18). Gold nanostructures, including gold nanorods (19), gold nanoshells (20), gold nanocages (21), and hollow gold nanospheres (22), have been most extensively studied as PTT reagents. Currently, the application of platinum (Pt) nanomaterials has attracted more and more attention in the biomedical field. Pt nanomaterials have extensive light absorption in the NIR region. For example, Manikandan et al. (23) reported for the first time that polyvinylpyrrolidone (PVP)-coated Pt nanoparticles (NPs) have photothermal conversion capability. However, this inorganic nanoplatform often faces problems of low biocompatibility when used in the biomedical field.

As a promising material for the preparation of multifunctional nanomaterials for cancer treatment, polydopamine (PDA) has attracted more attention in the biomedical field (24–26). Dopamine is a kind of biomimetic mussel adhesive protein, which can self-polymerize and spontaneously deposit onto various materials under alkaline pH conditions, forming a controllable PDA layer (27, 28). PDA coating has special adhesion ability and good biocompatibility (29). In addition, PDA, with a lot of catechol and amino groups on its outer surface, can contribute to nanomaterials’ functionalization of cell-specific ligands and biocompatibility (30). RGD peptide is related to tumor progression and less expressed in mature vascular endothelial cells and normal organ systems. Thus, RGD peptide has been used to modify nanomaterials to improve receptor-mediated cell adhesion by recognizing integrin α_v_β_3_ of cancer cells, thereby promoting endocytosis (31–33).

**Scheme 1:**
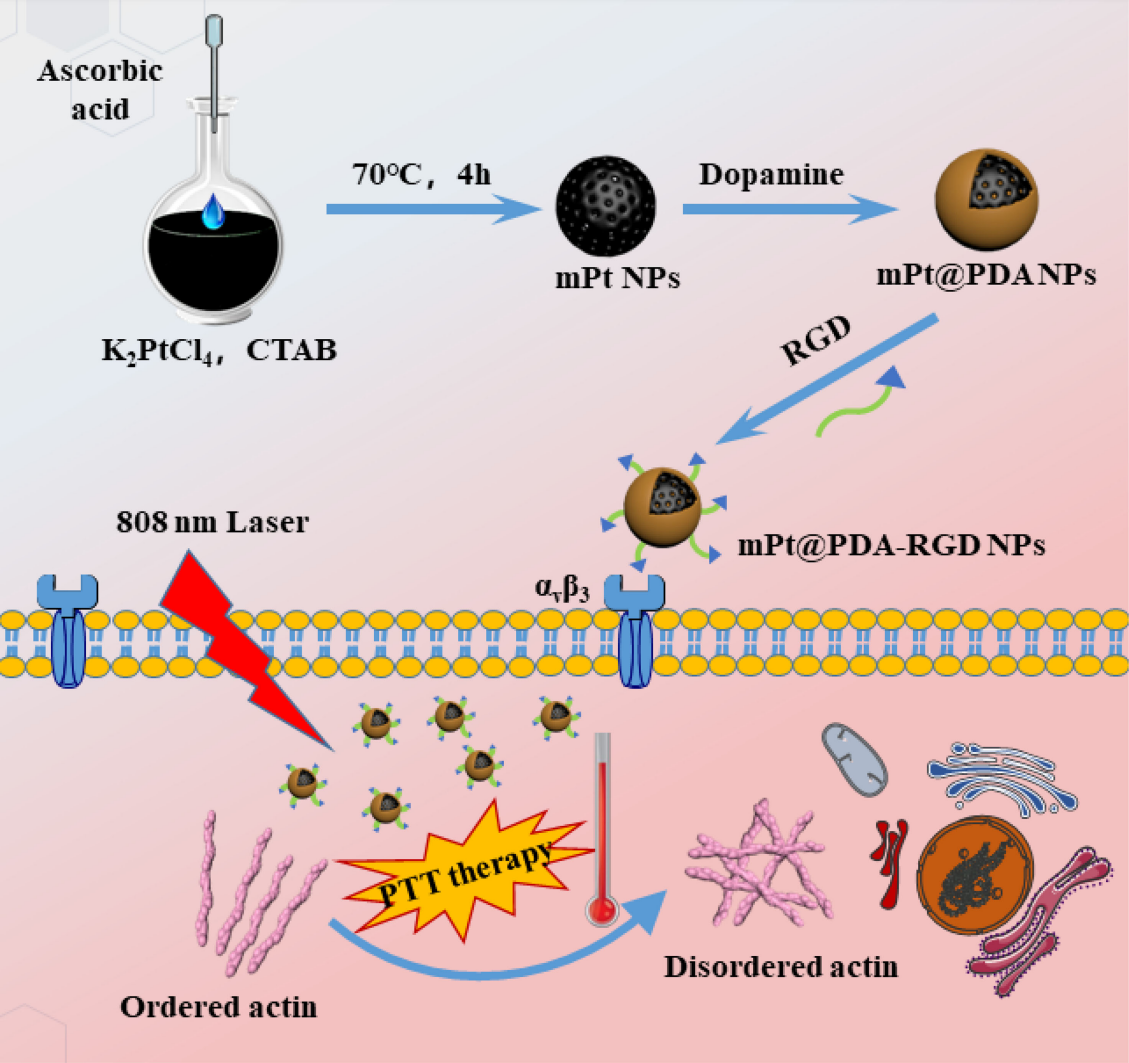
The synthesis of Arg-Gly-Asp peptide-coupled polydopamine-modified mesoporous platinum nanoparticles (mPt@PDA-RGD NPs) and their application in targeted cancer therapy.

In this work, Arg-Gly-Asp (RGD) peptide-coupled PDA-modified mesoporous Pt (mPt) (mPt@PDA-RGD NPs), with mPt as core, the self-polymerizing dopamine as the shell, and the grafted RGD as targeting ligands, were prepared (**Scheme 1**). Under 808-nm laser irradiation, mPt@PDA-RGD NPs exhibited excellent biocompatibility, chemotaxis of tumors, and efficient SKOV-3 inhibition ability. Immunofluorescence imaging showed that the actin cytoskeleton of SKOV-3 was destroyed and the migration of cancer cells was effectively inhibited after PTT. Thus, mPt@PDA-RGD NPs present great potential for targeted photothermal cancer treatments.

## Materials and Methods

### Materials

Potassium tetrachloroplatinate (II) (K_2_PtCl_4_), hexadecyl-trimethylammonium bromide (CTAB), ascorbic acid, dopamine hydrochloride (DA·HCl), and RGD were obtained from Aladdin. Tris-HCl buffer (10 mM, pH 8.5) was gained from Phygene (China). The cell counting kit-8 (CCK-8) was bought from Beyotime Biotechnology. Calcein AM and PI were obtained from KeyGen Biotech Co. (China). Cancer cell line SKOV-3 was obtained from the Type Culture Collection of the Chinese Academy of Sciences. Dulbecco’s modified Eagle’s medium (DMEM) was obtained from Procell (Wuhan, China). Penicillin-streptomycin, Fetal bovine serum (FBS), Trypsin-Ethylene Diamine Tetraacetic Acid (EDTA), and Phosphate buffered solution (PBS) were obtained from Solarbio Science & Technology Co. (China). All chemicals were purchased from commercial suppliers and directly used.

### Fabrication of mPt@PDA-RGD Nanocomposite

Pt NPs with porous structure were prepared *via* a simple chemical reduction process (34). Briefly, K_2_PtCl_4_ solution (10 mM, 36 ml) and CTAB solution (100 mM, 90 ml) were mixed together at 70°C for 0.5 h. Then, ascorbic acid (20 mM, 54 ml) was added to the mixture, and the solution was further heated at 70°C for 3 h. Excess CTAB was washed away with absolute ethanol; next, the nanoparticles were dispersed in deionized water. mPt@PDA NPs were synthesized by reaction of mPt NPs and dopamine in a 5:1 ratio in tris-HCl buffer (10 mM, pH 8.5) for 1 h. For the synthesis of the mPt@PDA-RGD, tris buffer (5 ml) containing RGD (10 mg) was mixed with mPt@PDA NPs (20 mg in 35 ml of tris-HCl buffer) for 30 min of ultrasonic treatment, followed by stirring for 12 h.

### Characterization

A high-resolution transmission electron microscopy (HRTEM;TecnaiG2 F20, FEI, Ohio, USA) was used to observe the morphology of mPt NPs and mPt@PDA NPs. The surface area and pore size of the mPt NPs were measured by using an automated gas sorption analyzer (Quantachrome, Autosorb-iQ). X-ray diffraction (XRD) pattern was obtained on a powder X-Ray diffractometer (Bruker, D8 ADVANCE). X-ray photoelectron spectroscopy (XPS) was performed using a multifunctional imaging electron spectrometer (Thermo ESCALAB 250XI). UV-Vis absorption spectra were taken on a U-3900 spectrophotometer (Hitachi, Japan). The hydrodynamic sizes and Z-potential were recorded by Malvern Zetasizer NanoZS90 (Malvern, United Kingdom). Fourier transform infrared spectroscopy (FTIR) spectra were tested on IRAffinity-1S (Shimadzu, Japan). Fluorescence imaging photographs were obtained by confocal laser scanning microscope (FV-3000, Olympus). The uptake of Pt element in SKOV-3 cells was measured by Inductively Coupled Plasma-Optical Emission Spectrometer (ICP-OES).

### Photothermal Properties of mPt@PDA-RGD Nanocomposite

To test the photothermal properties of nanomaterials, 0.2 ml of mPt@PDA-RGD NP solution with various concentrations (0, 25, 50, 100, 150, 200 μg/ml) was treated with 808-nm laser at power densities of 1.5 W/cm^2^ for 5 min. In addition, mPt@PDA-RGD NP solution (0.2 ml) of equal concentration (100 μg/ml) was treated with 808-nm lasers (0.5, 1,1.5, and 2 W/cm^2^). To study the thermal stability, 0.2 ml of mPt@PDA-RGD NP solution (100 μg/ml) was irradiated at 1.5 W/cm^2^ for four ON/OFF cycles. The corresponding temperatures are recorded per second with an infrared thermometer (CEM, DT-8891E).

### Cytotoxicity Test

To evaluate the toxicity of mPt NPs and mPt@PDA-RGD NPs by CCK-8 assay, first, SKOV-3 cells were seeded into a 96-well plate (1 × 10^4^ cells per well) overnight. Then, the cells were treated with mPt NPs or mPt@PDA-RGD NPs at 0, 10, 20, 40, 80, and 100 μg/ml in the cell incubator for 24 h. After washing with PBS, CCK-8 solution (10 μl) accompanied by culture medium (100 μl) was added to each well. After 2-h incubation without light, the absorbance was measured at 450 nm. Each experiment had 3 parallel groups.

### *In Vitro* Cellular Uptake Measurement

To investigate the cellular uptake of mPt@PDA-RGD NPs, SKOV-3 cells were transferred to a 6-well plate (1 × 10^5^ cells per well). After 24-h incubation, the old medium was replaced with fresh medium accompanied by mPt@PDA NPs or mPt@PDA-RGD NPs; both of their concentrations were 25 μg/ml. After 2-h or 4-h incubation, the cells were washed with PBS, treated with trypsin, and digested with 5 ml of *aqua regia*. Finally, Pt content in each sample was tested by ICP-OES. Each experiment was performed three times.

### Photothermal Therapy of Cancer Cells

In simple terms, SKOV-3 cells were grown on 96-well plates (5 × 10^3^ cells per well). There were four groups of the phototoxicity tests (control, laser, mPt@PDA-RGD NPs, and mPt@PDA-RGD NPs+laser). The control group was left untreated. The cells in the laser group were irradiated under 808-nm NIR (1.5 W/cm^2^) for 5 min. The remaining two groups received mPt@PDA-RGD NPs (50 μg/ml) and incubated for 24 h. Next, the cells were respectively irradiated without or with 808-nm laser (1.5 W/cm^2^) for 5 min. Finally, the viabilities of SKOV-3 cells were examined by CCK-8 kit.

### Cytoskeleton Staining

SKOV-3 cells were treated with mPt@PDA-RGD NPs (0, 50, 100 µg/ml) and irradiated with 808 nm (1.5 W/cm^2^) for 50 s, then fixed with 4% paraformaldehyde 15 min, permeabilized with Triton X-100 (0.2%), and blocked with 5% Bovine Serum Albumin (BSA) at room temperature for 1 h. Then washed with PBS and incubated with TRITC Phalloidin (MKbio, Shanghai, China) for 1 h and further stained by Hoechst 33342 (10 μl; 10 μg/ml). The labeled slides were mounted with Prolong™ Gold Antifade Reagent with 4',6-Diamidino-2-phenylindole Dihydrochloride (DAPI) (Invitrogen, Carlsbad, USA). The cells were imaged by using confocal laser scanning microscope (CLSM; FV-3000, Olympus, Japan).

### Western Blot

SKOV-3 cells were incubated with mPt@PDA-RGD NPs (0, 50, 100, 200 µg/ml) for 6 hours, then irradiated with 808 nm (1.5 W/cm^2^) for 50 s. After washing with cold PBS, cell lysate (Servicebio, China) and protease inhibitor were used to lyse the cancer cells, followed by incubation for 30 min at 4°C. Then, proteins were extracted by centrifuging. The extracted proteins were separated by 10% sodium dodecyl sulfate - polyacrylamide gel electrophoresis (SDS-PAGE) (Epizyme, China). Then, the proteins were transferred to polyvinylidene fluoride (PVDF). The membranes were incubated with primary antibodies (actin 1:2,000, glyceraldehyde-3-phosphate dehydrogenase (GAPDH) 1:1,000) at 4°C for 12 h. After washing three times, the membranes were incubated with secondary antibodies for 1 h, then washed with PBST, and the membranes were treated with chemiluminescence agents.

### Cell Migration Assay

SKOV-3 cells were inoculated in culture inserts to create a clean cell gap, and mPt@PDA-RGD NPs at a concentration of 50 μg/ml or 100 μg/ml were added to the holes when the cells have grown to 90% to form a single layer and incubated for 6 h, then cleaned with PBS, and treated with 808 nm (1.5 W/cm^2^) for 50 s. Cell migration was monitored 24 h after culture.

### Annexin V/ Propidium Assay

Annexin V/PI kit was utilized to determine apoptosis and necrosis rate. SKOV-3 cells were grown in 24-well plates and incubated for 12 h. The mPt@PDA-RGD NPs were added and incubated with the cells for 24 h. Then, the medium was refreshed and exposed to 808-nm laser (1.5 W/cm^2^) for 5 min. The cells were digested with trypsin and dispersed with binding buffer. After Annexin V and PI staining, the fluorescence intensity of cells was observed by flow cytometer (BD Accuri™ C6 Plus).

### *In Vitro* Photothermal Induced Cell Death

SKOV-3 cells were grown in glass bottom dishes. The mPt@PDA NPs or mPt@PDA-RGD NPs (100 μg/ml) were added, and cells were incubated for 4 h. Next, the cells were washed with PBS and refreshed with new medium and treated with 808-nm laser (1.5 W/cm^2^) for 5 min. Medium was removed, and 200 μl of Calcein AM/PI working solution was added for 30 min to stain cells. After careful cleaning with PBS, living and dead cells were analyzed by CLSM.

### Statistical Analysis

All results are presented as mean ± standard deviation (SD), and between-group comparisons were evaluated using one-way ANOVA. A p value < 0.05 indicates statistical significance, and data are represented as * for p < 0.05, ** for p < 0.01, *** for p < 0.001, and **** for p < 0.0001. NS stands for not statistically significant.

## Results and Discussion

### Preparation and Characterization of mPt@PDA-RGD Nanoparticles

**Figure 1:**
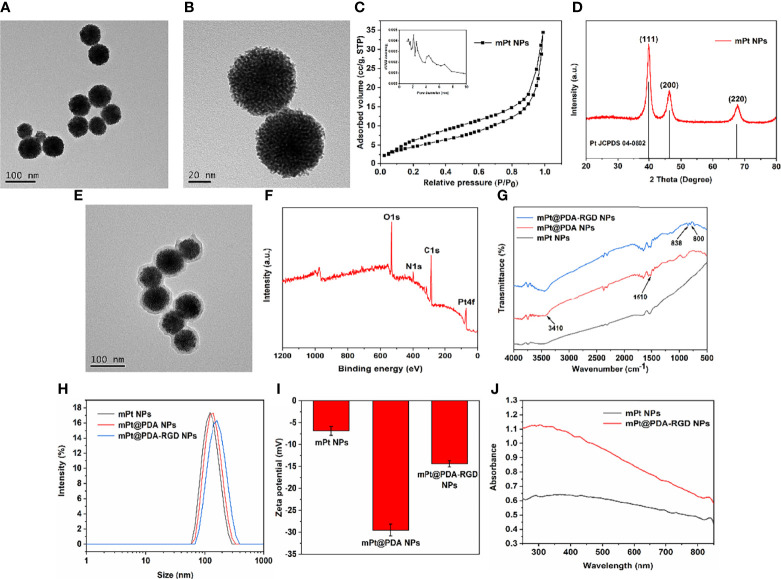
Transmission Electron Microscope (TEM) images of mPt NPs **(A, B)**. Pore size distribution **(C)** and X-ray diffraction (XRD) patterns **(D)** of mPt NPs. **(E)** TEM image of mPt@PDA NPs. X-ray photoelectron spectroscopy (XPS) spectra **(F)** of mPt@PDA NPs. **(G)** Fourier transform infrared spectroscopy (FTIR) of mPt@PDA NPs and mPt@PDA-RGD NPs. Size distribution **(H)** and Zeta potential **(I)** of mPt NPs, mPt@PDA NPs, and mPt@PDA-RGD NPs. **(J)** Uv-visible absorption spectrum (UV-Vis) spectral of mPt NPs and mPt@PDA-RGD NPs.

TEM image (**Figure 1A**) shows that the average diameter of the synthesized mPt NPs was about 70 nm, and the morphology of the synthesized mPt NPs was obviously porous (**Figure 1B**). The mesoporous shape was further confirmed by N_2_ absorption–desorption. As shown in **Figure 1C**, mPt NPs displayed wide pore diameter distribution (about 2–8 nm) and high surface area (46.76 m^2^/g). The XRD pattern of the mPt NPs (**Figure 1D**) was highly consistent with that of bulk Pt (JCPDS 04-0802). After PDA coating, the prepared mPt@PDA NPs showed a core/shell morphology (**Figure 1E**), and XPS spectra (**Figure 1F**) of mPt@PDA NPs further confirmed the presence of Pt elements. As shown in **Figure 1G**, the wide absorption bands at 3,410 cm^-1^ could be attributed to the stretching vibration of phenolic O-H and N-H. The bands at 1,510 cm^-1^ corresponded to shearing vibration of N–H. The above absorption bands on mPt@PDA NPs indicated the successful polymerization of PDA. Meanwhile, the new peaks of mPt@PDA-RGD NPs at 800 and 838 cm^-1^, which belonged to the peaks of phenyl group of RGD, confirmed the successful grafting of RGD. The diameter of NPs in aqueous solution was determined by Dynamic Light Scattering (DLS) method. The results (**Figure 1H**) showed that the average hydrodynamic diameter of mPt NPs, mPt@PDA NPs, and mPt@PDA-RGD NPs was about 124.5, 133.9, and 151.6 nm, respectively. Zeta potential (**Figure 1I**) shows that PDA-modified mPt NPs significantly reduced the potential, which was due to the hydroxyl group on the surface of PDA. The potential of mPt@PDA-RGD NPs was increased compared with that of mPt@PDA NPs, which was attributed to the positively charged amino group on RGD. UV-vis absorption spectra (**Figure 1J**) showed that mPt@PDA-RGD NPs had extensive absorption at the NIR region, confirming the good potential of mPt@PDA-RGD NPs as effective photosensitizers.

### Photothermal Effect Evaluation of mPt@PDA-RGD Nanoparticles

**Figure 2:**
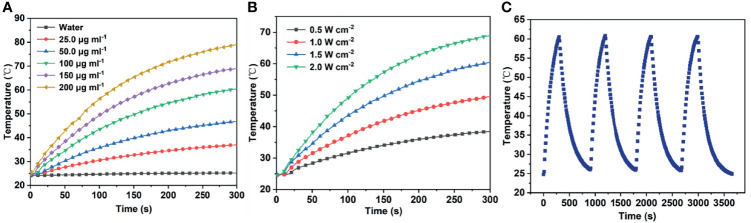
**(A)** Temperature responses of mPt@PDA-RGD NP solutions with various Pt concentrations under the 808-nm laser. **(B)** Temperature responses of mPt@PDA-RGD NP solutions (100 μg/ml) under the 808-nm laser with different power densities for 300 s. **(C)** The heating curve of the mPt@PDA-RGD NP dispersion in deionized water at 4 laser on/off cycles (1.5 W/cm^2^) under 808-nm laser irradiation.

Various concentrations of mPt@PDA-RGD NP aqueous solution were irradiated with 808 nm (1.5 W/cm^2^, 5 min); meanwhile, the temperature of samples was monitored. **Figure 2A** shows that the temperature increased with the increase of nanomaterial concentration. In contrast, the temperature of H_2_O was almost constant under the same irradiation conditions. Under a fixed concentration of mPt@PDA-RGD NPs (100 μg/ml), the change of temperature was positively correlated with the optical density (**Figure 2B**). In general, the increase of NP temperature showed the dependence of solution concentration and optical density. In addition, we evaluated the photothermal stability of the NPs by performing four cycles of “laser-on/off” experiments, during which mPt@PDA-RGD NPs were irradiated for 5 min and cooled naturally. **Figure 2C** shows after 4 cycles that the temperature did not decrease obviously, indicating that NPs had good photothermal stability. Thus, mPt@PDA-RGD NPs show excellent photothermal properties under 808-nm laser irradiation.

### Cellular Uptake Experiment

**Figure 3:**
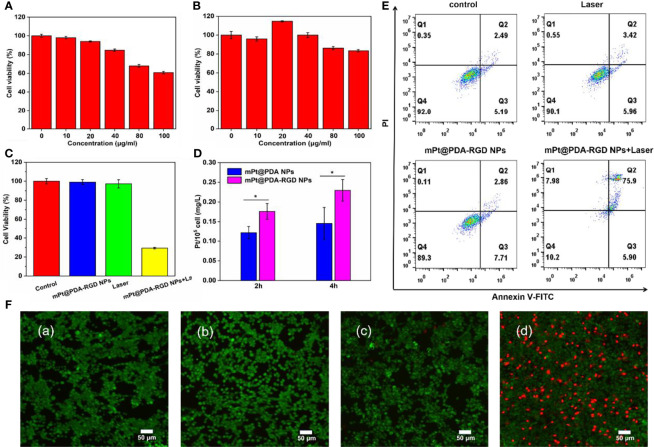
Cytotoxicity of **(A)** mPt NPs and **(B)** mPt@PDA-RGD NPs. **(C)** Relative viability of SKOV-3 cells treated with mPt@PDA-RGD NPs and laser. **(D)** Pt contents in SKOV-3 cells with different treatments. All values are presented as means ± standard errors of the mean (SEM); *p < 0.05. **(E)** Flow cytometry analysis of SKOV-3 cells. **(F)** Calcein AM/PI staining of SKOV-3 cells with various treatments: (a) control, (b) laser (808 nm), (c) mPt@PDA-RGD NPs, (d) mPt@PDA-RGD NPs+laser.

SKOV-3 cells were coincubated with mPt@PDA or mPt@PDA-RGD NPs for different times to detect the concentration of Pt internalization by ICP-OES. As shown in **Figure 3D**, the absorption of Pt elements was of positive relevance with the incubation time of NPs and SKOV-3 cells. In addition, more Pt elements were detected in cells treated with mPt@PDA-RGD NPs than in those treated with mPt@PDA NPs. These proved that mPt@PDA-RGD NPs have effective targeting ability to SKOV-3 cells.

### *In Vitro* Photothermal Therapy Effects of mPt@PDA-RGD Nanoparticles

To evaluate the cytotoxicity of mPt NPs and mPt@PDA-RGD NPs, CCK-8 assay was used to evaluate the cell viability, and the results displayed that mPt NPs showed poor biocompatibility at high concentrations (**Figure 3A**); however, after modification by PDA coating, mPt@PDA-RGD NPs had no significant toxicity on SKOV-3 cells under the concentration of 100 μg/ml (**Figure 3B**). Even when reaching 100 μg/ml, the viability of SKOV-3 cells was still above 80%, indicating that mPt NPs had obvious toxicity to cells, but the modification of PDA greatly reduced the toxicity of mPt NPs and improved the cytocompatibility of the NPs. We also tested the cytotoxicity of mPt@PDA-RGD NPs under PTT. **Figure 3C** shows no obvious cytotoxicity in the control group, laser group, and mPt@PDA-RGD NP group. However, the cell survival rate of the mPt@PDA-RGD NPs+laser group was reduced to about 29%, which reflected the excellent photothermal performance of mPt@PDA-RGD NPs.

Flow cytometry was further performed to quantify cell viability. mPt@PDA-RGD NPs (50 μg/ml) were cultured with SKOV-3 cells for 24 h. Next, cells were washed two times with PBS, new medium was refreshed, and cells were exposed to 808-nm laser irradiation; Annexin V and PI double staining was used to detect cytotoxicity. **Figure 3E** shows that the cell viability of the control, laser, and mPt@PDA-RGD NP groups was maintained at about 90%. However, the cell viability dropped to 10.2% in the mPt@PDA-RGD+laser group. The results show that mPt@PDA-RGD NPs can induce apoptosis significantly under NIR laser and is a highly efficient photothermal agent.

Moreover, Calcein AM/PI staining was used to further verify the PTT efficiency of mPt@PDA-RGD NPs. SKOV-3 cells in the control group, laser group, and mPt@PDA NP group showed bright fluorescent green, indicating the survival status of cells. However, the mPt@PDA-RGD NPs with laser group observed the most red fluorescence (**Figure 3F**), suggesting that PTT was effective in killing cancer cells.

**Figure 4:**
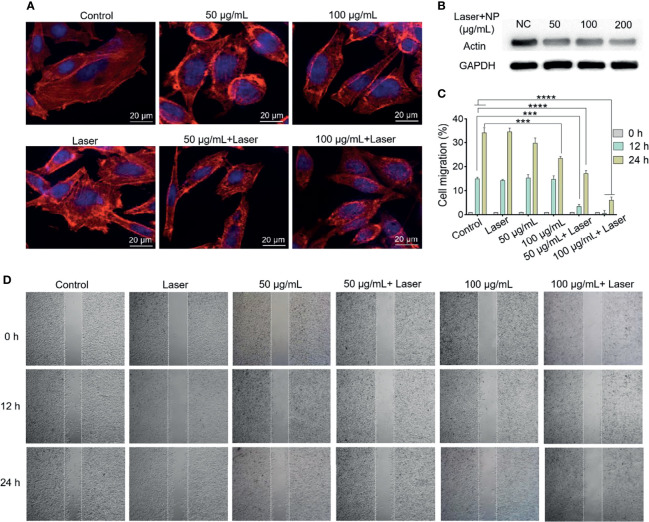
**(A)** Immunofluorescence images and **(B)** Western blot analysis of SKOV-3 actin staining after various treatments. **(C)** Corresponding migration rate. All values are presented as means ± standard errors of the mean (SEM); ****p < 0.0001, ***p < 0.001. **(D)** The migration performance of SKOV-3 cells with various treatments.

The effects of mPt@PDA-RGD NPs and PTT on actin cytoskeleton in SKOV-3 cells were further analyzed by CLSM. As shown in **Figure 4A**, the actin filaments in the cytoplasm of the control group showed highly ordered cytoskeleton arrangement with thick bundle distribution. The actin filaments in cytoplasm showed no obvious change after laser treatment, indicating that only laser treatment had almost no influence on the cytoskeleton. Interestingly, SKOV-3 cells with mPt@PDA-RGD NP treatment showed partial curling of the actin filaments at the cell edge, and the curling degree of actin filaments displayed obvious mPt@PDA-RGD NPs dose-dependent manner. When SKOV-3 cells were synchronously treated with mPt@PDA-RGD NPs and laser, the fluorescence intensity of actin filaments was obviously weakened, and the structure of F-actin contracted and curled seriously, losing the ordered arrangement state. Western blot analysis was further performed to verify the change of actin expression. As shown in **Figure 4B**, the expression of actin in SKOV-3 cells was significantly downregulated with the increase of mPt@PDA-RGD NP dose under the same laser intensity. These results indicated that the photothermal effect produced by mPt@PDA-RGD NPs+laser treatment can effectively disturb the cytoskeleton.

Subsequently, cell scratch experiments were used to investigate the antimigration performance of mPt@PDA-RGD NPs under laser. As shown in **Figures 4C, D**, there was no significant difference in the cell migration between the NIR group and the control group at 24 h, indicating that only laser irradiation had no significant effect on inhibiting migration. While the mPt@PDA-RGD NP group displayed a dose-dependent SKOV-3 cell migration inhibition, which was attributed to the disturbance of cytoskeleton by mPt@PDA-RGD NPs. With laser irradiation treatment, the cell migration of the mPt@PDA-RGD NP group was significantly reduced both at 12 and 24 h, indicating that the photothermal effect of mPt@PDA-RGD NPs produced a significant inhibitory effect on the cell migration of SKOV-3 cells.

PTT has been widely used in basic research and preclinical field. There are a few studies using porous Pt nanoparticles for PTT, and some limitations exist. For example, Zhu et al. (35) synthesized porous Pt nanoparticles coated with poly (diallyl dimethyl ammonium chloride) for PTT. More than 70% of the cells were phagocytosed by photothermal irradiation of 808-nm laser at 8.4 W/cm^2^ for 3 min. The photothermal effect is obvious, but the high laser power greatly limits the clinical application of the material. The mPt@PDA-RGD NPs synthesized by us have obvious photothermal effect under 808-nm laser at 1.5 W/cm^2^, which can effectively kill most tumor cells at a relatively safe power. Moreover, we found that the photothermal effect can destroy the actin cytoskeleton and inhibit tumor migration, which is rarely reported. This study provides a new direction for PTT of tumors.

## Conclusions

In conclusion, we developed a multifunctional PTT nanoplatform based on mPt@PDA-RGD NPs. As a photosensitizer, mPt was the core of NPs, and its surface was coated with PDA, which improves biocompatibility and facilitates functional modification of NPs.

Next, mPt@PDA NPs with RGD coupling can target highly expressed integrin α_v_β_3_ on SKOV-3 cells through receptor-mediated targeting. Cell experiments showed that mPt@PDA-RGD NPs can not only effectively induce tumor ablation but also greatly inhibit the migration ability of surviving cancer cells, which was attributed to the disturbance of actin cytoskeleton under the photothermal effect. Thus, mPt@PDA-RGD NPs showed great potential as an effective PTT nanomedicine for tumors targeted by photothermal ablation and migration inhibition.

## Data Availability Statement

The original contributions presented in the study are included in the article/supplementary material. Further inquiries can be directed to the corresponding authors.

## Author Contributions

JD: resources, supervision, project administration, and funding acquisition. XS: experiment design, supervision, validation, and funding acquisition. HW: conceptualization, methodology, resources, and supervision. XZ: formal analysis, data curation, visualization, and writing. GM: formal analysis, data curation, visualization, and writing. PZ: visualization and investigation. YC: visualization and investigation. All authors contributed to the article and approved the submitted version.

## Funding

This work was supported by the National Natural Science Foundation of China (22104073); Natural Science Foundation of Shandong (ZR2020MB073, ZR2021QB119); Shandong Health System Outstanding Young Talent Project, Overseas Science and Technology Talents Project of Shandong Province, Academic Promotion Program of Shandong First Medical University (2019QL008); and the Youth Outstanding Reserve Talents Program of Shandong First Medical University and Shandong Academy of Medical Sciences.

## Conflict of Interest

The authors declare that the research was conducted in the absence of any commercial or financial relationships that could be construed as a potential conflict of interest.

## Publisher’s Note

All claims expressed in this article are solely those of the authors and do not necessarily represent those of their affiliated organizations, or those of the publisher, the editors and the reviewers. Any product that may be evaluated in this article, or claim that may be made by its manufacturer, is not guaranteed or endorsed by the publisher.
